# First description of sarcoptic mange in a wild coati (*Nasua narica*), in Ecuador, and cooccurrence of canine distemper virus

**DOI:** 10.1590/S1984-29612022002

**Published:** 2022-01-14

**Authors:** Ricardo Villalba-Briones, Cristian Barros-Diaz, Abel Gallo-Pérez, Miquel Blasco-Carlos, Eliana B. Molineros

**Affiliations:** 1 ESPOL Polytechnic University, Escuela Superior Politécnica del Litoral – ESPOL, Facultad Ciencias de la Vida, Campus Gustavo Galindo, Guayaquil, Ecuador; 2 Fundación Proyecto Sacha, Guayaquil, Ecuador; 3 Fundación para la Conservación e Investigación JaPu, Guayaquil, Ecuador; 4 Escuela de Nutrición y Dietética, Universidad Espíritu Santo-Ecuador – UEES, Guayaquil, Ecuador; 5 Instituto de Investigación e Innovación de Salud Integral, Universidad Católica de Santiago de Guayaquil – UCSG, Guayaquil, Ecuador

**Keywords:** Sarcoptic mange, Nasua, Canine distemper virus, urbanization, wildlife, Sarna sarcóptica, Nasua, vírus da cinomose canina, urbanização, vida selvagem

## Abstract

We present a case of *Sarcoptes* and canine distemper virus (CDV) infection in a white-nosed coati (*Nasua narica*) that was trapped in the dry tropical forest of Cerro Blanco reserve, located in the coastal region of Ecuador. Sarcoptic mange is a highly contagious and zoonotic disease with worldwide distribution that causes epidemics. Mange is produced by *Sarcoptes* mites that causes severe epidermal damage. Secondary infections and physiological constrictions without treatment can lead to death of the host. In addition, cooccurrence of canine distemper virus was detected via iiRT-PCR from serum samples. Physical analyses showed that 90% of the skin was affected by severe alopecia due to the sarcoptic mange infection. The presence of mites and histopathological analyses confirmed the diagnosis of infection. This coati was taken to a veterinary clinic and was fed every day, but it died after four days. This is the first report of sarcoptic mange and the first report of CDV in white-nosed coatis in South America. Further studies are needed in this region, to seek out other suspected cases, given the high capacity for disease transmission. Preventive actions to avoid epidemic and zoonotic episodes are needed.

## Resumo

Apresentamos um caso de *Sarcoptes* e infecção pelo vírus da cinomose canina (CDV) em um quati-do-nariz-branco (*Nasua narica*) que ficou preso na floresta tropical seca da reserva de Cerro Blanco, localizada na região costeira do Equador. A sarna sarcóptica é uma doença altamente contagiosa e zoonótica de distribuição mundial que causa epidemias. A sarna é produzida por ácaro do gênero *Sarcoptes* que causa graves danos epidérmicos. Infecções secundárias e constrições fisiológicas sem tratamento podem levar à morte do organismo. Além disso, a coocorrência do vírus da cinomose canina foi detectada, via iiRT-PCR, a partir de amostras de soro. As análises físicas mostraram que 90% da pele estava afetada por alopecia severa devido à infecção pelo ácaro da sarna sarcóptica. A presença de ácaros e análises histopatológicas confirmaram o diagnóstico de infecção. Esse quati foi levado a uma clínica veterinária e foi alimentado todos os dias, mas morreu após quatro dias. Esse é o primeiro relato de sarna sarcóptica e o primeiro relato de CDV em quatis-de-nariz-branco na América do Sul. São necessários mais estudos nessa região, para buscar outros casos suspeitos, dada a alta capacidade de transmissão da doença. Ações preventivas para evitar episódios epidêmicos e zoonóticos, são necessárias.

## Introduction

Coatis, mammals catalogued in the order Carnivora, family Procyonidae and genus *Nasua*, are widely distributed throughout the Americas (Hass & Valenzuela, 2002). Their gregarious behavior makes them gather in groups of 2 to 26 individuals that may split into subgroups for hours or days depending on their biological needs and environmental opportunities (Hass & Valenzuela, 2002). Coatis exhibit a suit of behaviors; some of them do not necessarily include physical contact, such as foraging with the juveniles in the center of the group, sharing vigilance, alarm, but some of them necessarily involve physical contact, such as mobbing, predators-prey interactions, and grooming, which usually takes place between members of the same group calling (Hass & Valenzuela, 2002). Furthermore, apart from antagonistic behaviors, intergroup grooming has also been recorded in several species of *Nasua* (Hass & Valenzuela, 2002).

Mange is a highly contagious skin disease with worldwide distribution that is produced through infestation by microscopic mites of different species, most commonly *Sarcoptes scabiei* or *Notoedres cati* (Currier et al., 2011; Fuller, 2013). The *Sarcoptes* genus is monospecific and cosmopolitan, belongs to the family Sarcoptidae, order Sarcoptiformes, infects humans, domestic and wild animals. Adult mites use their mouthpieces and hooks on their legs to create burrows in the epidermis of the host. This causes alopecia, hyperkeratosis and erythema, and it is often accompanied by intense pruritus, which can be debilitating and produce a notable morbidity (Karimkhani et al., 2017). These epidermal issues often degenerate into secondary bacterial or fungal infections that produce weakening and loss of body mass which can also cause death (Karimkhani et al., 2017). The lack of protective fur and exposure also effects on the thermoregulatory system (Escobar et al., 2021).

Sarcoptic mange, also known as scabies, has been shown to have a severe impact on the survival of individuals of various species, and, also, may have considerable declining effect at community, populations and species level (Escobar et al., 2021). A GPS monitoring study on mange-infected raccoon dogs (*Nyctereutes procyonoides*) showed that they gradually decreased their activity levels and home range until they died after 32 to 52 days (Süld et al., 2017). Mange has been recorded in humans, camelids, ungulates, lagomorphs, didelphimorphs, chiropters and carnivores such as mustelids, canids or procyonids, among other wildlife taxa, reaching 12 orders, 39 families and 148 species of domestic and wild mammals (Montecino-Latorre et al., 2020; Verdugo et al., 2016; Zhao et al., 2015).

Although sarcoptic mange has been shown to have worldwide prevalence, few reports have been published about its presence in South American wild animals (Montecino-Latorre et al., 2020; Verdugo et al., 2016). Most reports have been done on domestic animals such as rabbits (*Oryctolagus cuniculus*), dogs (Canis *familiaris*), cats (*Felis catus*), pigs (*Sus domesticus*), cattle (*Bos taurus*), llamas (*Lama glama*) and alpacas (*Vicugna pacos*) (Alcaino & Gorman, 1999; Bornstein, 2010; Gonzalez-Astudillo et al., 2018). Epidemic episodes and associated high rates of mortality have been detected in different species of herbivores and carnivores on different continents, such as in lynxes (*Lynx rufus*) and American black bear (*Ursus americanus*) in North America (USA); foxes (*Vulpes vulpes* and *Vulpes macrotis* mutica) in Europe (Fennoscandia) and USA; and wombats (*Lasiorhinus* sp. and *Vombatus ursinus*) in Australia (Escobar et al., 2021).

Furthermore, mange has a human impact: in addition to current cases, a mange epidemic affected several communities in Ecuador from 1968 to 1973 (Carvajal et al., 1977; Karimkhani et al., 2017). Transmission occurs through direct contact through allogrooming, mating and territorial fights or through indirect contact with infected dens, resting areas, and burrows (Escobar et al., 2021). Even if several variant forms of *Sarcoptes scabiei* can coexist due to regionalism, they still are able to display their infection cycles both in humans and in diverse animal hosts (Zhao et al., 2015).

Domestic animals can become vectors, hosting *Sarcoptes* mites and transmitting mange to wildlife (Gonzalez-Astudillo et al., 2018). Because of the lack of a formal national monitoring system in Ecuador, tracking of wild animals health and diseases becomes a very difficult task. Apart from definitive histopathological and genetic analyses and diagnostic observations, photographic evidence of severe alopecia or hyperkeratosis in wild animals has been used to report suspected cases of mange (Montecino-Latorre et al., 2020).

In Ecuador, few reports of sarcoptic mange have been addressed academically. The Andean porcupine (*Coendou quichua*) has been found affected with mange (Gonzalez-Astudillo et al., 2018), but not procyonids. Remarkably, Valenzuela et al. (2000) reported for the first time an epizootic event of mange on wild *Nasua narica* in North America. However, in this case, it was caused by *Notoedres cati*, variant forms of *S. scabiei* have not been reported on wild white-nosed-coatis in South America, and neither have been reported in cooccurrence with Canine distemper virus.

Canine distemper disease (CD) is a highly contagious disease caused by a virus that affects both dogs and wild animals, leading to fatal consequences in Procyonidae, Canidae, Mustelidae, Delphinidae and Felidae, among other taxa (Michelazzo et al., 2020). It is caused by the canine distemper virus (CDV), which is a pleomorphic RNA virus that belongs to the genus *Morbillivirus* (family Paramyxoviridae) (Sykes, 2014). Presence of CDV can lead to severe multisystemic disease affecting primarily the gastrointestinal, respiratory, and neurological systems (Sykes, 2014). In wild animals of the genus *Nasua*, CDV has been documented as occurring in North America and Central America (Rodríguez-Cabo-Mercado et al., 2020; Rojas-Jiménez et al., 2021).

The present study provides the first report of sarcoptic mange infestation in wild animals in the coastal region of Ecuador (Cerro Blanco reserve), and the first report of sarcoptic mange in the genus *Nasua*, specifically on the white-nosed coati (*N. narica*), in South America. The study subject also manifested cooccurrence of CDV infection.

## Materials and Methods

The Cerro Blanco Protected Forest is located to the west of the city of Guayaquil (Guayas Province, Ecuador) (2° 10’ 50.15” S; 80° 01’ 01.93” W), near the southeastern extremity of the Chongón-Colonche mountain range, at an altitudinal range from 20 to 507 meters above sea level. The Cerro Blanco is a protected area of 6,078 hectares that is characterized as a dry forest part of the Tumbesian ecoregion, which covers from northern Peru to Colombia. In Ecuador, this ecoregion has suffered high anthropogenic impact and depletion of the vegetation cover (Cueva Ortiz et al., 2019). The study area has a rainy season from January to April, when 85% of the precipitation occurs (Cueva Ortiz et al., 2019). Thus, water scarcity stress becomes an issue for the wildlife during the long dry season.

**Figure 1:**
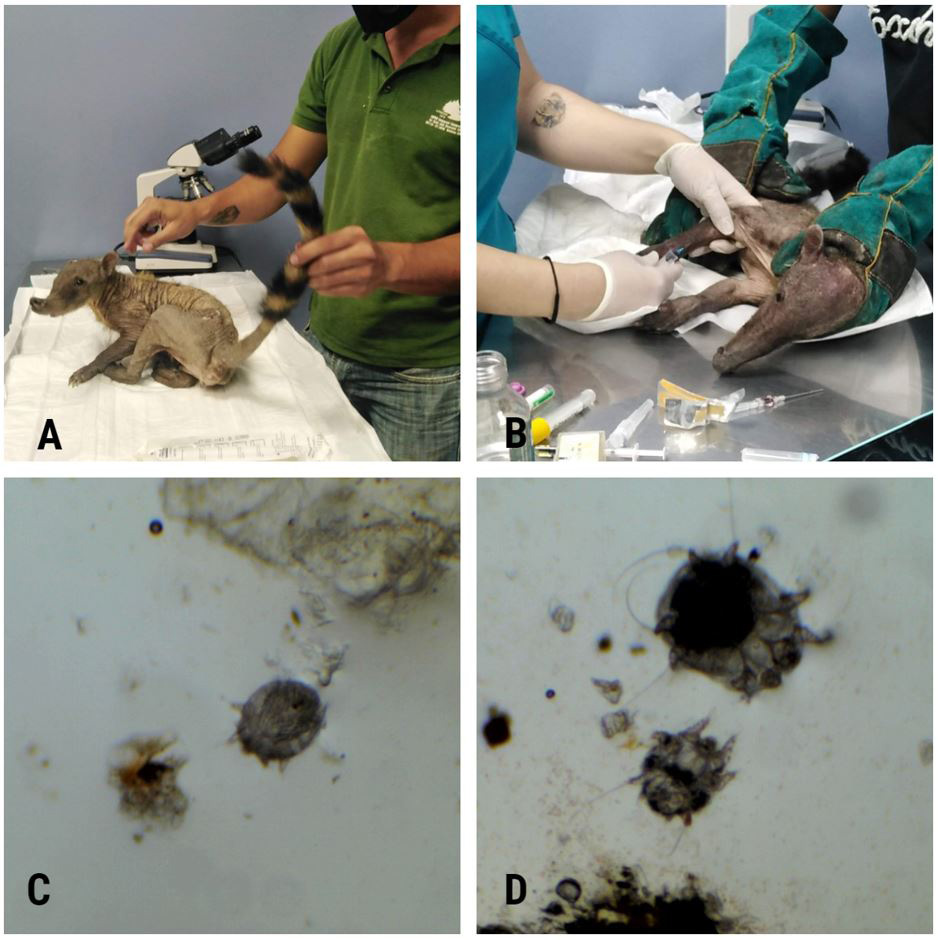
(A) White-nosed coati showing more than 90% of body surface covered by mange infection; (B) White-nosed coati (*Nasua narica*) undergoing medical treatment; (C) Mite and epithelial tissue from white nosed coati (*Nasua narica*); (D) Photograph of mites at different stages of development.

Several camera traps were installed in areas of special interest, such as river basins. Several species and individuals with skin abnormalities were identified, and therefore it was decided to conduct a trapping campaign. On December 19, 2020, a female white-nosed coati (*N. narica*) was caught from among a group made up of 12 individuals, composed of adult and juvenile females, in a camping zone in the forest. This individual had very little fur along its body (Figure 1A). It was caught by means of a Tomahawk trap (102 x 25 x 45 cm), lured with tuna. The trap was placed in an area of transitional dry forest at a distance of 20 m from the touristic picnic area (-2.182765; -80.016603).

After capture, this animal was taken to a private clinic with wildlife treatment permission from the Environmental Ministry of Ecuador (Mansion Mascota clinic, Guayaquil). It was medicated intramuscularly with ketamine (2 mg/kg) and acepromazine (1 mg/kg) in order to obtain blood and skin samples. The skin sample was obtained by scraping the skin using a scalpel. The samples were analyzed and sent to a private laboratory in Guayaquil (Diagnovet, Guayaquil) for confirmation. Mites of the Sarcoptidae family were rinsed with 2% potassium hydroxide and observed under a binocular microscope to identify the parasite (Kandi, 2017).

For mange treatment, 6 micrograms of Ivermectin® was applied subcutaneously. The coati was fed every day, but after a period of four days it died. Necropsy was carried out to extract samples from the heart, lung, liver and spleen and sent to a private laboratory (Histo-dx vet, Quito).

The blood samples were sent to a private laboratory in Guayaquil (Diagnovet) for an RNA-based Insulated Isothermal Reverse Transcriptase PCR” (iiRT-PCR) analysis to be conducted. After centrifugation, the serum samples were analyzed using iiRT-PCR POCKIT for CDV detection. For the iiRT- PCR analysis procedure, the POCKIT™ nucleic acid analyzer point-of-need PCR detection tool was used, following the instructions given by the manufacturer (Genereach Biotechnology, Taichung, Taiwan). The commercial POCKIT tool successfully combines advanced insulated isothermal polymerase chain reactions using primers designed by the manufacturer, in independent laboratories associated with research. Primers for the virus used by de POCKIT™ commercial kit were not provided by the producer. Genereach technical service argued confidential reasons not to provide primers sequence but informed about the target gene designed for the CDV detection, which is “N gene”.

## Results

Physical analyses showed that more than 90% of the body surface was covered by sarcoptic disease, i.e. all areas except for most of the tail and a thin line above the shoulder blades (Figure 1A). These lesions consisted of complete alopecia throughout the body, with crusts dispersed across the animal’s back. No clinical symptoms of CDV were diagnosed to the specimen.

*Sarcoptes* infection was collected in the tissue of the coati, through analysis of the epithelial tissue. Under an optical microscope, magnification 40x, it was possible to observe the characteristics of *Sarcoptes scabiei,* as shown by dichotomous key for the subfamilies and genera of Sarcoptidae (Fain, 1968). The mites (Figures 1C and 1D) morphology showed a slightly elongated but globose idiosoma with triangular denticles in the mid-dorsal area, parallel transverse striae, short and broad gnathosoma, short and thick legs with fused tibia and tarsus IV, ventral median apodeme fused with genital sclerites, anal shields in the male not fused to the posterior median shield, which are characteristics of *Sarcoptes scabiei*.

In addition, samples analyzed by the private laboratory (Diagnovet, Guayaquil, Ecuador) confirmed the results. Moderate quantities of eggs, larval forms and live adults of *Sarcoptes scabiei* were detected in the laboratory. No spore hairs or arthrospores were observed but there were abundant scaly epithelial cells and moderate amounts of cell debris. No inflammatory cells were observed. Moderate numbers of bacterial bodies (cocci) with Gram tinction were observed and there was a sensitive response to amx-clavulanate, cephalexin, cefoxitin, clindamycin, erythromycin and trimethoprim-sulfa.

**Figure 2:**
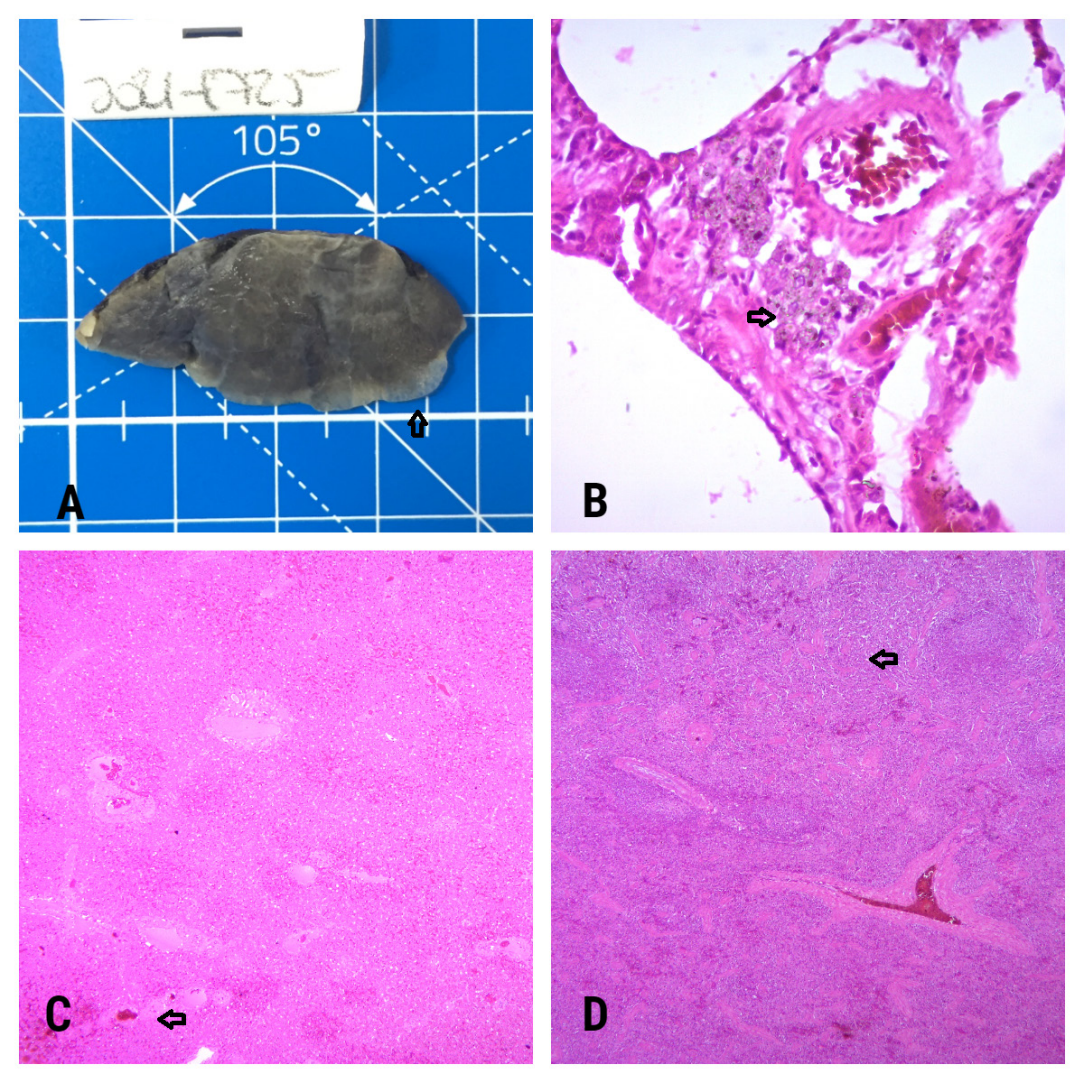
(A) Lung showing mild emphysema in the lower lobe; (B) Granular material in lung tissue; (C) Microscopic view of liver sample showing slight centrilobular congestion; (D) Microscopic view of spleen sample showing slight reduction of red pulp.

Analysis of the liver under a microscope showed slight centrilobular congestion (Figure 2C), and decreased amounts of red pulp were seen in the spleen (Figure 2D). However, no obvious pathological changes were found in the spleen, kidneys, and heart. The lungs showed dilation and rupture of the alveolar septa (emphysema) at the edge of the parenchyma and in the lower lobe (Figure 2A). Discrete aggregates of activated macrophages multifocally infiltrating the bronchiolar and bronchiolar connective tissue were observed, and refringent granular and crystalloid material (pneumoconiosis) were visible in their cytoplasm (Figure 2B).

In addition, the blood samples tested positive for canine distemper virus through the iiRT-PCR analysis.

## Discussion

This was the first report of sarcoptic mange in the white-nosed coati (*N. narica*) in South America. However, it had previously been detected in North and Central America (Valenzuela et al., 2000). Moreover, although studies on CDV in North and Central America have been published (Rodríguez-Cabo-Mercado et al., 2020; Rojas-Jiménez et al., 2021), this study was also the first record of CDV in this species in South America.

The individual that was captured showed severe (Escobar et al., 2021) and extensive infestation with *Sarcoptes*. that covered more than 90% of the body surface. The diagnosis from the histopathological analyses showed that there were no obvious pathological changes in the liver, spleen, kidneys and heart. The lungs presented mild, multifocal anthracosis, probably produced due to the dusty environment and terrestrial habits of the animal; and alveolar emphysema, produced probably due to the breathing on particulate soil during the animal’s foraging activities (March et al., 2000). No signs of CDV or other influence from mange was noticed in these analyses.

The impact of the disease on the individual depends generally on its health, genetic resistance, magnitude of the infection and length of time of exposure. Virulent disease can cause the death of the host and panzootic episodes (Süld et al., 2017; Valenzuela et al., 2000).

Coatis are social animals that live in groups and actively feed on gathered fruits and animals, in constant gregarious movement that favors contagion events (Valenzuela et al., 2000). Sarcoptic mange is more likely to be present when individuals are exposed to harsh environment conditions ( Escobar et al., 2021 ; Foley et al., 2016). In addition, dry-season water scarcity induces concentrations of fauna around the ponds of the Cerro Blanco protected area, which increases the likelihood of transmission.

Dispersion of the disease is related to the density of susceptible animals, presence of domestic animals, habitat quality and anthropogenic effect (Valenzuela et al., 2000). High-density urban populations and high numbers of uncontrolled domestic animals and feral dogs living in the vicinity of coatis’ habitats build up a probable line of transmission of incessant flux, which should be addressed (Valenzuela et al., 2000).

Secondary illnesses play an important role in leading to a fatal outcome (March et al., 2000; Valenzuela et al., 2000). In the case of this coati, cooccurrence of CDV seems to have reinforced both diseases. Although the detection of CDV was an incidental finding, we cannot rule out the possibility that CDV infection may have contributed to the coati’s clinical condition and subsequent death. Even if we cannot discard a deterioration of the nervous system in the CDV positive coati due to the lack of such analyses in the necropsy, no clinical signs related to CDV were identified in the individual.

Infected animals constitute reservoirs and vectors for diseases, with the ability to spread them. Mange is one of the diseases with highest prevalence among dogs in Ecuador (López et al., 2009). Moreover, the proximity of the forest to urban areas of Guayaquil and commonplace presence of dogs in the reserve suggest that transmission of *Sarcoptes* infection to wildlife from domestic animals has become facilitated.

## Conclusion

This first report on sarcoptic mange in the white nosed coati in South America confirms its presence in the Cerro Blanco reserve. Given the high capacity for mange infection in the coati population and the high diversity of possible hosts, the risk of sarcoptic mange epidemy in wild communities is palpable in the Cerro Blanco reserve and other forest areas near Guayaquil. Furthermore, just like sarcoptic mange infection, cooccurrence of CDV implies another threat that could originate through contact with domestic animals and need to be addressed locally and regionally. This confirmation of a case of mange plus CDV in white-nosed coatis emphasizes the need for further studies in the area and, moreover, indicates the need for intervention with preventive actions and dog population management, in order to protect wildlife health and impede zoonotic transmission to the neighboring human populations.
